# RNA-Seq and Gene Ontology Analysis Reveal Differences Associated With Low R/FR-Induced Shade Responses in Cultivated Lentil and a Wild Relative

**DOI:** 10.3389/fgene.2022.891702

**Published:** 2022-06-20

**Authors:** Hai Ying Yuan, Carolyn T. Caron, Albert Vandenberg, Kirstin E. Bett

**Affiliations:** ^1^ Department of Plant Sciences, University of Saskatchewan, Saskatoon, SK, Canada; ^2^ Aquatic and Crop Resource Development Research Center, National Research Council of Canada, Saskatoon, SK, Canada

**Keywords:** shade, RNA-seq, hormones, transcription factors, *Lens culinaris*, *Lens orientalis*

## Abstract

Lentil is an important pulse crop not only because of its high nutrient value but also because of its ecological advantage in a sustainable agricultural system. Our previous work showed that the cultivated lentil and wild lentil germplasm respond differently to light environments, especially to low R/FR-induced shade conditions. Little is known about how cultivated and wild lentils respond to shade at the level of gene expression and function. In this study, transcriptomic profiling of a cultivated lentil (Lupa, *L. culinaris*) and a wild lentil (BGE 016880, *L. orientalis*) at several growth stages is presented. *De novo* transcriptomes were assembled for both genotypes, and differential gene expression analysis and gene ontology enrichment analysis were performed. The transcriptomic resources generated in this study provide fundamental information regarding biological processes and genes associated with shade responses in lentils. BGE 016880 and Lupa shared a high similarity in their transcriptomes; however, differential gene expression profiles were not consistent between these two genotypes. The wild lentil BGE 016880 had more differentially expressed genes than the cultivated lentil Lupa. Upregulation of genes involved in gibberellin, brassinosteroid, and auxin synthesis and signaling pathways, as well as cell wall modification, in both genotypes explains their similarity in stem elongation response under the shade. Genes involved in jasmonic acid and flavonoid biosynthesis pathways were downregulated in BGE 016880 only, and biological processes involved in defense responses were significantly enriched in the wild lentil BGE 016880 only. Downregulation of WRKY and MYB transcription factors could contribute to the reduced defense response in BGE 016880 but not in Lupa under shade conditions. A better understanding of shade responses of pulse crop species and their wild relatives will play an important role in developing genetic strategies for crop improvement in response to changes in light environments.

## Introduction

Plants have developed a variety of strategies to respond to environmental stresses, including the detection of their neighbors through changes in light quality. Preferential absorption of the red spectrum in green plants leads to the reduction in the red to far-red ratio (R/FR) in dense vegetation. The R/FR change is sensed by phytochromes and signals the close presence of neighboring plants as potential competition, thereby inducing a complex adaptive response known as the shade avoidance syndrome (SAS). Typical SAS characteristics include increased stem elongation, reduced branching, accelerated flowering, reduced biomass, decreased leaf number, and reduced yield (Franklin, 2008; Green-Tracewicz et al., 2012; Chitwood et al., 2015; Gong et al., 2015).

Molecular components and gene networks controlling SAS have been extensively studied and reviewed in *Arabidopsis* and a few model plants (Casal, 2012; Ballaré and Pierik, 2017; Fernández-Milmanda and Ballaré, 2020). Phytochromes are the family of photoreceptors that respond to the R/FR part of the spectrum and exist in two photoconvertible isoforms: an inactive Pr form that is triggered by R light and an active Pfr form that is induced by FR light (Quail, 2002; Wang, 2015). Photoconversion of phytochromes triggers downstream signaling networks that regulate subsequent responses (Vandenbussche et al., 2005; Legris et al., 2019). Phytochromes can also directly interact with multiple transcription factors, such as basic helix–loop–helix (bHLH) transcription factors, to regulate responses to light signals (Ballaré and Pierik, 2017). Furthermore, various plant hormones such as auxin, gibberellic acid (GA), cytokinin (CK), ethylene, and brassinosteroid (BR) have been reported to be involved in SAS (Kurepin et al., 2007; Kozuka et al., 2010; Iglesias et al., 2018; Fernández-Milmanda and Ballaré, 2020). Shade also affects jasmonic acid (JA)- and salicylic acid (SA)-mediated plant immune system and reduces volatile JA levels (Moreno et al., 2009; De Wit et al., 2013; Kegge et al., 2013; Fernández-Milmanda et al., 2020). The balance between SAS and plant immunity and the crosstalk among the pathways involved have been intensely studied and reviewed (Huot et al., 2014; Rea, 2019; Pierik and Ballaré, 2021).

Despite concentrated studies of SAS in model plants, little is known about it in legumes. A study on phenological and growth of annual medics (*Medicago* spp.) and clovers (*Trifolium* spp.) in response to shading showed increased crop height, delayed flowering, reduced soil coverage, and aboveground dry biomass in these species (Mauro et al., 2014). Root nodules and the expression of JA-responsive genes were reduced under low R/FR light in *Lotus japonicus* (Suzuki et al., 2011). Typical SAS responses such as long internodes, early flowering, and reduced branching exhibited by *Lotus japonicus* were linked to the induction of transcription factors such as *LjHB2*, a homeodomain (HD) leucine zipper (ZIP) family protein, and *LjIAA29*, an auxin response factor (Ueoka-Nakanishi et al., 2011). Upregulation of *msPIF3* and *msHB2* under low R/FR light contributed to the SAS in alfalfa (*Medicago sativa*) (Lorenzo et al., 2019). A previous study from our group revealed that both wild and cultivated lentils showed typical SAS-like increased shoot elongation and longer internodes under low R/FR light, but the responses to flowering time and seed yield varied (Yuan et al., 2017).

Low R/FR-related shade conditions are typical in natural environments where there is a high density of vegetation such as crop fields with high seeding rates or with high weed pressure. The lentil is known to be a poor competitor with weeds, and understanding how it responds to competition can help us breed for cultivars that respond better under dense canopy conditions. In addition, a better control of SAS in legume crops such as lentil may lead to higher yield when increasing plant density to feed the increasing world population and meet protein need while reducing its land-use footprint. To better manipulate SAS in lentils, it is necessary to understand the molecular components and regulatory network controlling this phenomenon in this species. By altering levels of R/FR, we can simulate shade and examine responses in different genotypes. In this study, the lentil response was examined through a time-series transcriptome profiling and differential gene expression (DEG) analysis in a genotype of cultivated lentil (*L. culinaris*) and a wild *L. orientalis* genotype exposed to high and low R/FR conditions. GO annotation analysis of *de novo*-constructed transcriptomes and GO enrichment analysis of DEGs were used to understand biological processes involved in low R/FR-related shade responses between the two species. Combining this with the *L. culinaris* genome assembly (CDC Redberry, v2.0) (Ramsay et al., 2021), we were able to identify shade-responsive genes, transcription factors, and their respective enriched GO biological processes involved in this important response.

## Materials and Methods

### Plant Material, Library Preparation, and Sequencing

*L. culinaris* cv. Lupa and *L. orientalis* accession BGE 016880, which have shown diverse responses to changes in light quality (Yuan et al., 2017), were used in this study. An Apogee spectroradiometer (Apogee Instruments, Model PS-300, Logan, UT, United States) was used to measure the spectral photon flux and the photosynthetically active radiation (PAR) in two Conviron walk-in plant growth chambers used in the study. The spectral photon flux at 650–670 and 720–740 nm was used to calculate R and FR values (Smith, 1982). Growth chambers fitted with T5 835 High Output fluorescence bulbs (Philips, Andover, MA, United States) had a high R/FR ratio of 7.30 ± 0.14 with the PAR at 402.2 ± 33.6 µmol/m2s. Evenly spaced PfrSpec™ LED light panels (Fluence Bioengineering, Inc., Model RAY44, peak spectrum at 730nm, Austin, Texas, United States) were added into the light bank to reach a simulated shade condition with the R/FR ratio of 0.19 ± 0.01 with the PAR at 395.6 ± 32.9 µmol/m2s. Both chambers had contrasting different R/FR ratios but similar light quantities based on the PAR. The optimum temperatures of 22°C/16-h day and 16°C/8-h night were used in both environments to grow the plants.

Leaf samples used for RNA-seq were collected 2 weeks after emergence; then, the leaf sample collection was continued once a week for 5 weeks. The growth stages of leaf samples in 5 weeks were referred to as stages T1 through T5, respectively. Samples were collected at the same time of the day, and three biological replicates were used with leaf materials harvested from three individual plants in each biological replicate. RNeasy Plant Mini Kit (Qiagen, Germantown, MD, United States) and on-column DNase digestion were used to extract total RNA, according to the kit instruction. NanoDrop 8000 UV–Vis spectrophotometer (NanoDrop, Wilmington, DE, United States) and Agilent RNA 6000 Nano Assay through an Agilent 2100 Bioanalyzer (Agilent Technologies, United States) were used to check the quantity and quality of the extracted RNA. Illumina TruSeq Stranded mRNA Sample Preparation Kit (Illumina Inc., San Diego, CA, United States) was used to prepare RNA-seq libraries. Libraries of groups of 20 barcoded samples were pooled, and paired-end sequencing (2 × 125 bp) was performed on an Illumina HiSeqTM 2500 system (Illumina Inc., San Diego, CA, United States).

### *De Novo* Assembly of the Transcriptome and Functional Gene Annotation

FastQC version 0.11.9 (Andrews, 2010) was used to check the quality of raw reads, and Trimmomatic version 0.38 (Bolger et al., 2014) was used for quality trimming to remove adaptor sequences. After checking overrepresented sequences and the adaptor content from the FastQC reports, we chose to use the TruSeq3-PE-2.fa file, one of the adaptor sequence files supplied by the Trimmomatic for trimming the adaptor. We kept the minimum read length to be 45 with a phred score of 33 in the trimming step. FastQC was run again to check the data quality, and the adaptor content was acceptable for the next step. After trimming, more than 97% of the sequences were kept paired for downstream analysis. All trimmed paired read data of 30 RNA-seq samples (5 stages, 2 light environments, and 3 bio-reps) from *L. culinaris* cv. Lupa were used to assemble the Lupa transcriptome using Trinity version 2.8.4 (Grabherr et al., 2011), and the same applied to *L. orientalis* BGE 016880 for the BGE 016880 transcriptome. We used default settings of Trinity for the *de novo* transcriptome assembly except that an increase in the CPU to 40 and the maximum memory to 150G was set to speed up the process to deal with the large data set. The assembly statistics were examined using the TrinityStats perl script available from Trinity utilities. Transcript abundance was then estimated using the Salmon package version 0.12.0 (Patro et al., 2017) and the align_and_estimate_abundance perl script from Trinity utilities for all 30 samples each of Lupa and BGE 016880 using their respective *de novo*-assembled transcriptomes. SuperTranscripts that contain the sequence of all exons of a gene without redundancy were constructed from *de novo* transcriptomes of both genotypes using Lace version 1.14.1 (Davidson et al., 2017) for downstream analysis. Transcripts encoding fewer than 67 amino acids were filtered out from the data set before functional gene annotation (Zhang, 2000), and the functional gene annotation of the transcriptomes was processed using FunctionAnnotator (Chen et al., 2017).

### Analysis of Differentially Expressed Genes

Differential gene expression was assessed using 3D RNA-seq pipeline version 2.0.0 (Calixto et al., 2018; Guo et al., 2020). The details are as follows: ten factor groups (T1.HighRFR, T2.HighRFR, T3.HighRFR, T4.HighRFR, T5.HighRFR, T1.LowRFR, T2.LowRFR, T3.LowRFR, T4.LowRFR, and T5.LowRFR) were included within the RNAseq data set with three biological replicates for each in both genotypes resulting in 60 samples in total. The lengthScaledTPM method from the tximport R package version 1.10.0 (Soneson et al., 2016) was used to generate read counts per million (CPM) and transcripts per million reads (TPMs) with inputs of transcript quantifications generated from Salmon version 0.12.0 (Patro et al., 2017) in the previous step. Transcripts with count per million reads (CPM) ≥ 1 in at least one of the 30 samples were identified as expressed, and a gene was considered expressed if any of its transcripts was expressed. Batch effects were estimated using the RUVSeq R package, version 1.16.0, with RUVr approach (Risso et al., 2014), and gene read counts across samples were normalized to log_2_ CPM using the trimmed mean of M-values (TMM) method (Robinson and Oshlack, 2010). The limma R package was then used for differential expression comparison (Law et al., 2014; Ritchie et al., 2015). Gene expression changes under the low R/FR light quality environment were examined using the high R/FR light quality environment from the corresponding time point as the control. For the T1 stage, differential gene expression groups were named Lupa_T1_LowHigh and BGE_T1_LowHigh, and the contrast groups were set as (T1.LowRFR-T1.HighRFR) for both *L. culinaris* cv. Lupa and *L. orientalis* BGE 016880. To examine the overall expression changes under the low R/FR light quality environment when compared to the high R/FR light quality environment, the contrast group was set as ((T1+T2+T3+T4+T5) LowRFR/5 and (T1+T2+T3+T4+T5) HighRFR/5) for both *L. culinaris* cv. Lupa and *L. orientalis* BGE 016880 and named as Lupa_LowHigh and BGE_LowHigh. Log_2_ fold change (Log_2_FC) represented the log_2_ CPM value differences in contrast groups for differential expression, and *p*-values from multiple testing were adjusted with a BH procedure to correct the false discovery rate (FDR) (Benjamini and Hochberg, 1995). A gene was determined to be differentially expressed in a contrast group if it had an adjusted *p*-value < 0.05 and |Log_2_FC| ≥ 1. These differentially expressed genes (DEGs) would more likely be genes involved in shade avoidance syndrome (SAS) at respective stages/conditions.

### GO Functional Enrichment Analysis of DEGs

TopGO, version 2.36.0 (Alexa and Rahnenfuhrer, 2020), was used for GO enrichment analysis of DEGs using the gene annotation results from the FunctionAnnotator (Chen et al., 2017) obtained in the previous step. The analysis was performed using the ParentChild algorithm (Grossmann et al., 2007). GO terms with fewer than five annotated genes were excluded from the analysis. Overrepresentation of GO terms within the group of DEGs was derived from Fisher’s exact tests, and a *p*-value < 0.05 was used to define the significantly enriched GO terms for the input DEG set.

### Transcription Factor Families and Their Presence in DEGs

Mercator pipeline, version 4.0 (Lohse et al., 2014; Schwacke et al., 2019), was used to identify TFs from *de novo*-assembled transcriptomes of both genotypes. TFs were classified using transcription factor families that were identified primarily using the Plant Transcription Factor Databases PlnTFDB and PlantTFDB as a guide (Pérez-Rodríguez et al., 2009; Jin et al., 2017). The identified TFs were then used to check for their presence among the DEGs.

### DEG Identification in the *L. culinaris* Reference Genome

To connect the DEGs obtained from the *de novo* assemblies of BGE 016880 and Lupa transcripts with genes in the *L. culinaris* reference genome, we used an in-house-developed perl script (Supplementary Material S1) to rename the DEGs based on their alignments with the *L. culinaris* v2.0 genome (Ramsay et al., 2021). This provided a candidate gene list (Supplementary Material S3) for further investigating genes involved in SAS in lentils. DEGs that had multiple alignments with the reference genome were not included in the list. TF DEGs of interest were further subjected to NCBI BLASTx search (https://blast.ncbi.nlm.nih.gov/Blast.cgi), and the max. alignment score and the percent sequence identity with the e-value cut-off of le-5 were used to select the homologs.

## Results

### *De Novo* Assembly of the Transcriptome and Functional Gene Annotation

**FIGURE 1:**
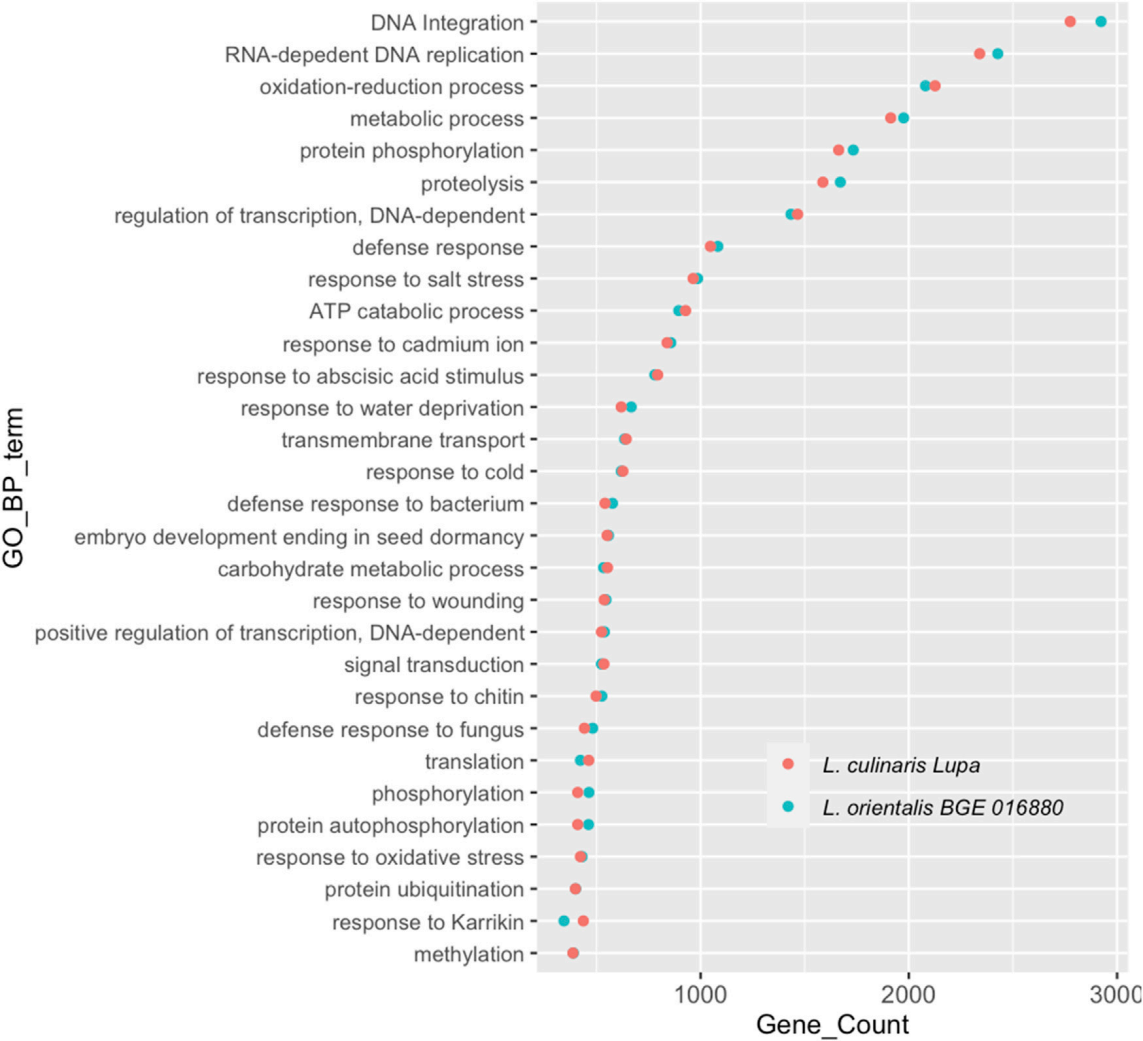
Top 30 gene ontology terms in the biological process (GO-BP) assigned to genes from *de novo*-assembled transcriptomes of *L. orientalis* BGE 016880 and *L. culinaris* cv Lupa. *X*-axis represents correspondent number of genes for each GO-BP term.

A *de novo* assembly approach was used for both BGE 016880 and Lupa RNA-sequencing data to avoid any bias introduced by mapping transcripts to the reference assembly genotype that is a different cultivar than Lupa and a different species than BGE 016880. The *de novo* assembled transcriptomes of both genotypes shared a high similarity regarding the GC percentage, contig N50, and median contig length, with only 2%–3% difference in the overall transcript and gene numbers. Assembled transcripts were annotated using FunctionAnnotator (Chen et al., 2017), and overall statistics are presented in Table 1. More than 88% of the filtered transcripts were annotated in both genotypes. The top five species homologs of these annotated genes were all legumes and corresponded to 84.1 and 83.9% of the annotated genes in BGE 016880 and Lupa, respectively (Supplementary Material S2-Supplementary Figure S1). Gene numbers from the top 30 GO-BP terms annotated in both genotypes were similar (Figure 1), and the top five GO-BP terms identified included “DNA integration,” “RNA-dependent DNA replication,” “oxidation–reduction process,” “metabolic process,” and “protein phosphorylation” in both genotypes.

**TABLE 1:** Summary statistics of genes and their annotations from *de novo*-assembled transcriptomes of *L. orientalis* BGE 016880 and *L. culinaris* cv. Lupa. Total annotated genes, the GO-BP ID, and terms were obtained through FunctionAnnotator (Chen et al., 2017), while transcription factors were identified using the Mercator pipeline (Lohse et al., 2014; Schwacke et al., 2019).

	*L. orientalis* BGE 016880	*L. culinaris* cv. Lupa
Total annotated genes	46,038	44,722
Genes with gene ontology: biological process (GO-BP) ID	27,071	26,258
GO-BP term identified	9,087	8,993
Transcription factors identified	1,622	1,593

### Differential Gene Expression Analysis and GO Enrichment Analysis of DEGs

Differentially expressed genes were determined by comparing expression levels under low relative to high R/FR conditions, that is, the changes that occur under shade-like conditions, in each genotype separately. Upregulated genes are those that were higher under low R/FR than under high R/FR, and downregulated genes are those that were lower under low R/FR. A total of 933 and 434 DEGs were obtained from the samples at the T1 stage for BGE 016880 and Lupa, respectively. When group means from the five development stages were used to look at the gene expression under the low R/FR-induced shade condition, 297 and 156 DEGs were identified from BGE 016880 and Lupa, respectively. BGE 016880 had more DEGs than Lupa in general, and DEGs from the T1 stage were more than those from the group mean of all five stages in both genotypes. Only 144 and 30 DEGs were shared between DEGs from the T1 stage and from the group mean of all five stages for BGE 016880 and Lupa, respectively (Supplementary Material S2-Supplementary Figure S2).

**FIGURE 2:**
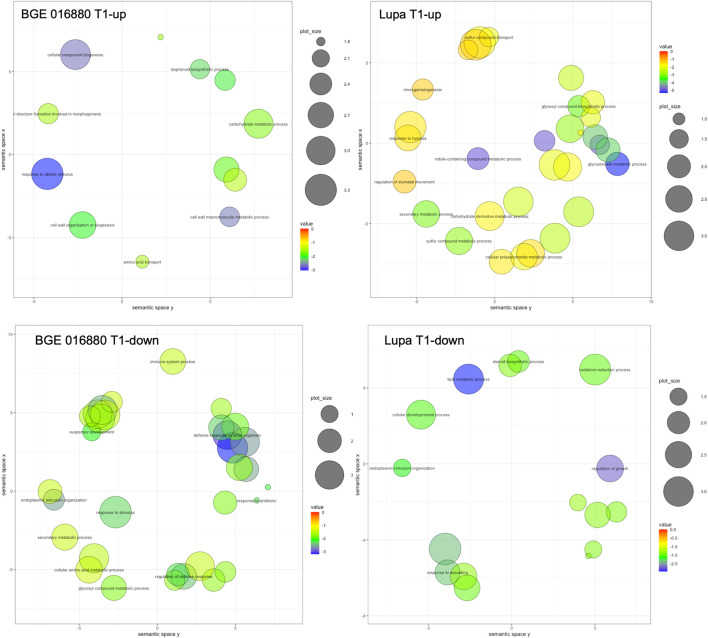
Scatter plots show representative GO-BP processes from GO enrichment analysis of DEGs at the T1 stage under low R/FR-induced shade condition for both *L. orientalis* BGE 016880 and *L. culinaris* cv Lupa. The plots were generated using REVIGO (Supek et al., 2011), and the representative GO terms within the clusters were shown in the plots. Bubble color indicates the *p*-value of the enrichment analysis resulted from TopGO, and only GO terms with significant *p*-values (*p*-value ≤ 0.05) were included in generating the scatter plot. Size indicates the frequency of the GO term in the gene ontology database, and more general GO terms are larger in bubble size.

To gain insight into the potential biological meaning of the large DEG sets obtained, we further used GO enrichment analysis to classify their function with a focus on the GO category of “biological process” (GO-BP). In total, 12 and 31 GO-BP terms were significantly enriched in upregulated DEGs from the T1 stage in BGE 016880 and Lupa, respectively, with processes such as “carbohydrate metabolic process” shared by both genotypes (Figure 2 and Supplementary Material S2-Supplementary Figure S3). “Response to abiotic stimulus,” “cellular component biogenesis,” and “isoprenoid biosynthetic process” were among the top enriched GO-BP terms in BGE 016880, while “glucosinolate metabolic process” and “indole-containing compound biosynthetic process” were among the top enrichment list in upregulated DEGs from the T1 stage in Lupa. A total of 33 and 18 GO-BP terms were significantly enriched in downregulated DEGs from the T1 stage in BGE 016880 and Lupa, respectively, with “endoplasmic reticulum organization” shared by both genotypes (Figure 2 and Supplementary Material S2-Supplementary Figure S4). GO-BP terms such as “regulation of defense response” and “defense response to other organism” were significantly enriched in downregulated DEGs from the T1 stage in BGE 016880 but not in Lupa.

Upregulated DEGs from all five stages in BGE 016880 were associated with a significant enrichment of 30 GO-BP terms, while in Lupa, there were 36 terms (Supplementary Material S2-Supplementary Figure S3). Among these, “floral whorl development” was among the top GO-BP terms shared between the two genotypes. Downregulated DEGs in BGE 016880 were associated with the significant enrichment of 44 GO-BP terms but only 12 in Lupa (Supplementary Material S2-Supplementary Figure S4). “Organic cyclic compound biosynthetic process” and “endoplasmic reticulum organization” were among the GO-BP terms shared between BGE 016880 and Lupa. GO-BP terms such as “cellular response to osmotic stress,” “response to water deprivation,” and “jasmonic acid-mediated signaling pathway” were significantly enriched in downregulated DEGs in BGE 016880 but not in Lupa.

### Transcription Factors in *De Novo* Assembled Transcriptomes and DEGs

**FIGURE 3:**
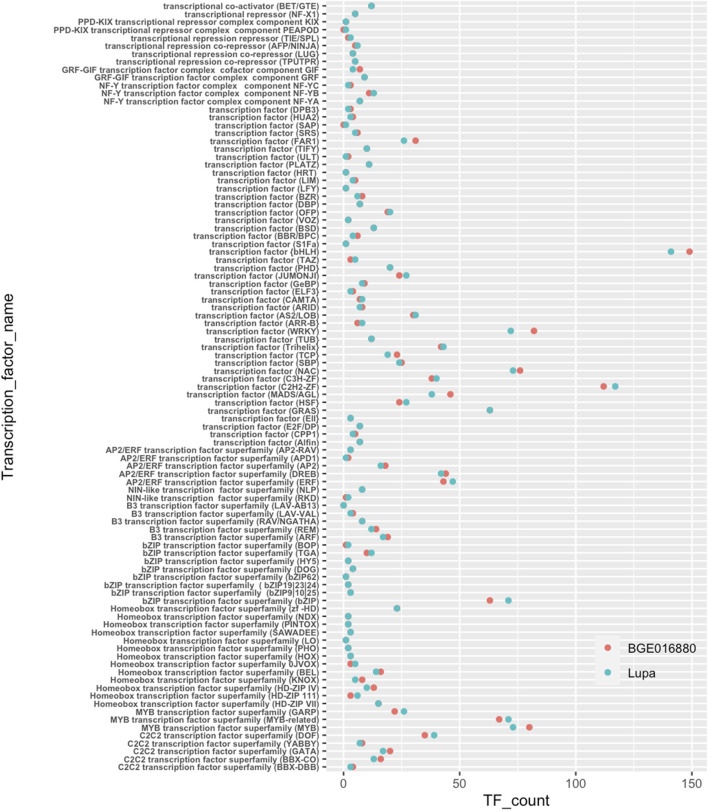
Transcription factors annotated from *de novo*-assembled transcriptomes of *L. orientalis* BGE 016880 and *L. culinaris* cv Lupa. *X*-axis represents correspondent numbers of transcription factors from each category.

Transcription factors (TFs) have been shown to be a major player in light-regulated transcriptional networks such as SAS (Jiao et al., 2007; Buti et al., 2020). Therefore, we specifically looked at transcription factors for their potential involvement in SAS in lentils. Similar numbers and categories of TFs were annotated for both genotypes (Figure 3 and Table 1). The basic helix–loop–helix (bHLH) was the largest TF class in both genotypes with 149 belonging to BGE 016880 and 141 to Lupa. Besides bHLH, C2H2-ZF, bZIP, MYB, NAC, and WRKY were other larger transcription factor classes identified in both genotypes.

We then used the generated TF list to look at TF presence in DEG sets. At the T1 stage, there were four times more TF DEGs identified in BGE 016880 than in Lupa (Supplementary Material S2-Supplementary Table S1). Of these 84 TF DEGs in BGE 016880, 64 were downregulated, with the majority coming from bHLH, MYB, WRKY, NAC, and homeobox transcription factors. In contrast, 14 of 21 TF DEGs in Lupa were downregulated at the T1 stage and belonged to the bHLH, MYB, AP2/ERF, and B3 classes of TFs. There were 43 TF DEGs in BGE 016880 from the group mean of all five stages but only 20 in Lupa (Supplementary Material S2-Supplementary Table S1). The regulation of MADS/AGLs was similar in both genotypes; however, as a large TF class, WRKY had good representation within TF DEGs in BGE 016880 but not in Lupa.

### DEG Identification Using the *L. culinaris* Reference Genome

**FIGURE 4:**
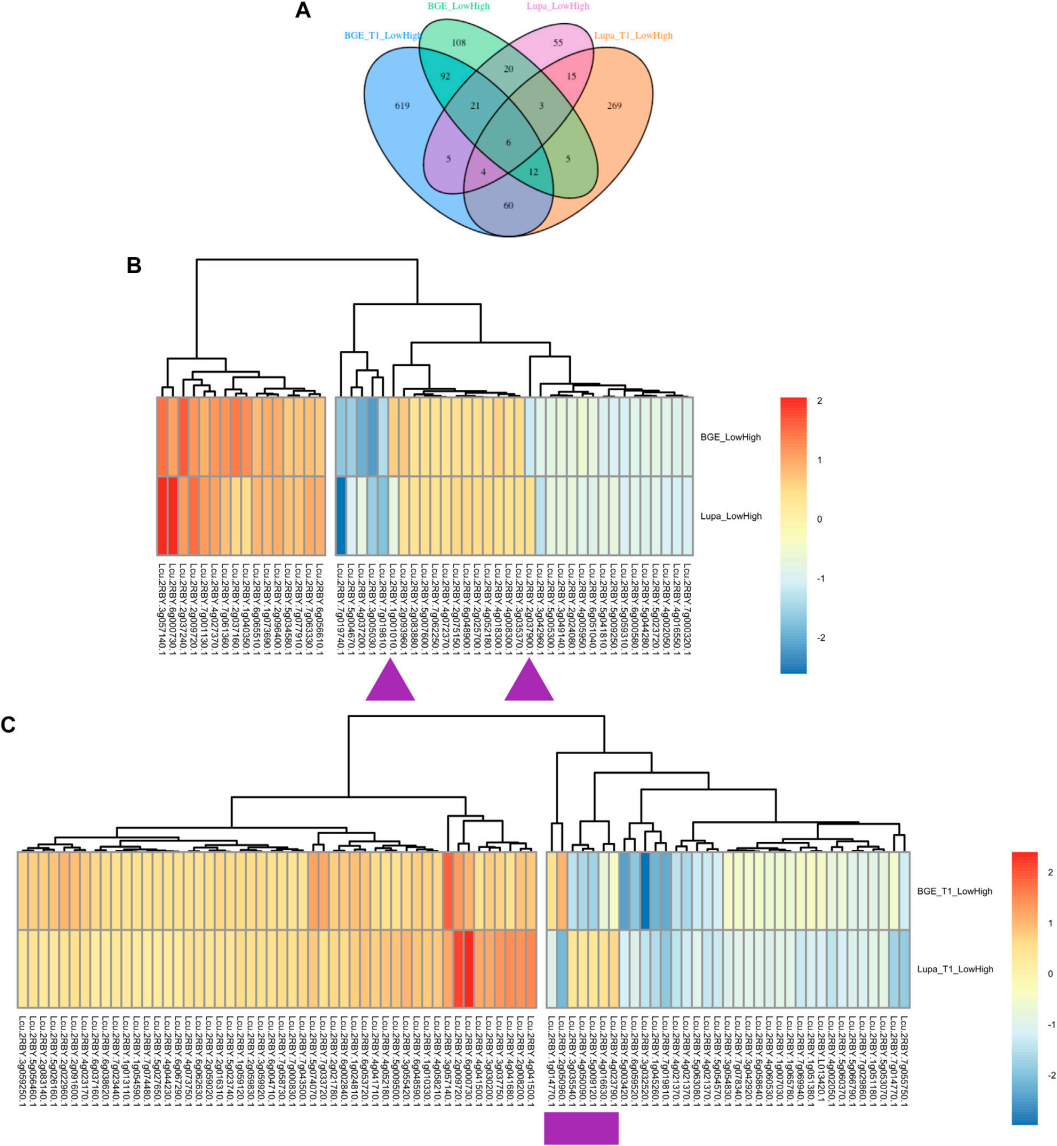
**(A)** Venn diagram showing numbers of unique and common differentially expressed genes (DEGs) under low R/FR-induced shade conditions between the T1 stage and all five stages for both *L. orientalis* BGE 016880 and *L. culinaris* cv. Lupa after renaming the DEGs based on their alignments with the *L. culinaris* v2.0 reference genome (Ramsay et al., 2021). **(B)** Heatmap shows expression trends of common DEGs from all five stages for *L. orientalis* BGE 016880 and *L. culinaris* cv. Lupa. **(C)** Heatmap shows expression trends of common DEGs from the T1 stage for *L. orientalis* BGE 016880 and *L. culinaris* cv. Lupa. Log fold changes of DEGs were transformed into z-score for heatmap generation. Purple arrows/rectangle underneath the genes referred to the opposite regulation of DEGs between two genotypes. Gene numbers were from the *L. culinaris* v2.0 reference genome based on alignments of these DEGs with the reference genome.

To connect these DEGs obtained from the *de novo* assemblies of both BGE 016880 and Lupa with the genes in the *L. culinaris* reference genome, we renamed the DEGs based on their alignments to the *L. culinaris* v2.0 reference genome. A DEG list with the corresponding gene number and gene description from the *L. culinaris* v2.0 reference genome for each set of DEGs was generated (Supplementary Material S3) and used to ensure orthologs were being compared. Clear differences were shown by development stages, as well as by species (Figure 4A). There were only six common DEGs shared by all four groups (BGE_T1_LowHigh, BGE_LowHigh, Lupa_T1_LowHigh, and Lupa_LowHigh). Of 50 common DEGs from the group mean of all five stages for both BGE 016880 and Lupa, only two genes showed opposite regulation (upregulated vs. downregulated), while the majority showed similar regulation patterns but different expression fold changes between BGE 016880 and Lupa (Figure 4B and Supplementary Material S3). Seven genes showed opposite regulation among the 82 common DEGs at the T1 stage for both BGE 016880 and Lupa (Figure 4C and Supplementary Material S3).

Within the DEGs that were shared by all four groups, four genes were consistently upregulated: a GA 20 oxidase gene (Lcu.2RBY.3g057140) that is involved in the biosynthesis of GA; two flowering locus T genes (FTb1/b2, Lcu.2RBY.6g000730 and Lcu.2RBY.6g000760) that are known to be involved in flowering time regulation; and a gene belonging to the MYB transcription factor family (Lcu.2RBY.2g009720). Upregulated genes at the T1 stage for both genotypes included genes involved in auxin signal transduction small auxin-up RNAs (*SAURs*, Lcu.2RBY.4g041680 and Lcu.2RBY.4g044230), a GA-stimulated transcript (GAST, Lcu.2RBY.6g048590), and genes involved in cell wall modification (Lcu.2RBY.1g010330, Lcu.2RBY.2g022960, and Lcu.2RBY.7g058730). However, genes involved in flavonoid biosynthesis such as chalcone and stilbene synthase family protein (Lcu.2RBY.3g046440) and chalcone synthase 6 (Lcu.2RBY.5g001660) genes involved in jasmonic-acid synthesis such as lipoxygenase (Lcu.2RBY.4g036980), and WRKY transcription factors (Lcu.2RBY.3g029190 and Lcu.2RBY.4g062520) were among the downregulated DEGs in BGE 016880 only at the T1 stage. These gene descriptions were in line with the results from the GO enrichment analysis of DEGs from the T1 stage for both genotypes.

Genes belonging to the MADS-box transcription factor family such as agamous-like genes (Lcu.2RBY.1g040350, Lcu.2RBY.2g037240, and Lcu.2RBY.7g063330) and bHLH transcription factor family genes (Lcu.2RBY.6g065510 and Lcu.2RBY.7g001130) were among those upregulated DEGs from the group mean of all five stages in both genotypes (Supplementary Material S4). Nevertheless, genes involved in jasmonic-acid synthesis such as lipoxygenase (Lcu.2RBY.4g036980) and WRKY transcription factors (Lcu.2RBY.3g029190, Lcu.2RBY.3g064420, Lcu.2RBY.4g062520, and Lcu.2RBY.6g033120) were again among the downregulated DEGs in BGE 016880 only from the group mean of all five stages. These gene descriptions were again in line with the results from the GO enrichment analysis.

## Discussion

Shade avoidance syndrome (SAS) includes various interrelated responses to changes in the light environment, and a wide range of components act simultaneously to contribute to the overall responses. Significant progress has been made in understanding the molecular regulation and gene networks controlling SAS in *Arabidopsis*; however, there is limited information available in legume crops such as lentils. Integration of knowledge obtained from *Arabidopsis* and crop plants will benefit breeders wanting to manipulate SAS effects for crop improvement under field conditions with varying light environments. One of the main triggers of SAS is a change in light quality, primarily a reduction in the red to far-red ratio. By changing this ratio in controlled growth chambers, we can simulate the effect of shading on plants while controlling for confounding effects such as competition for other resources. Results from our earlier lentil study (Yuan et al., 2021) suggested potential roles for genes within the florigen gene family, several MADS-box transcription factors, and bHLH transcription factors, in regulating flowering time under such conditions in lentils. In this study, we used a holistic approach to examine the gene expression changes under different light qualities, as well as gene functionalities between the cultivated lentil and its wild relative, *L. orientalis*, to help better understand SAS in lentils.

### High Degree of Similarity Exists in Transcriptomes Between Wild *L. orientalis* and Cultivated *L. culinaris*

*L. orientalis* is generally accepted as the wild progenitor of *L. culinaris* (Ladizinsky, 1999; Sonnante et al., 2003). A study of 60 accessions from across the genus *Lens* using genotyping-by-sequencing (GBS) method further confirmed that *L. orientalis* is genetically most closely linked to *L. culinaris* (Wong et al., 2015). The phylogenetic tree constructed using SNP data obtained from the GBS method showed that the species boundary between these two is difficult to distinguish due to the paraphyletic relationship (Wong et al., 2015).

*Lens* species have different genome sizes, and chromosomal rearrangements have been observed from an earlier karyotype study (Ladizinsky, 1979), as well as our recent research efforts (Gela et al., 2021; Ramsay et al., 2021). Of the two genotypes used in our study, BGE 016880 is a *L. orientalis* accession, while Lupa is a *L. culinaris* cultivar from Spain (Fratini et al., 2007). Using the *L. culinaris* reference genome, which is based on the Canadian cv. CDC Redberry, for transcriptome assembly and downstream differential gene expression analyses could bias the results. Therefore, a *de novo* assembly approach was used for both BGE 016880 and Lupa RNA-sequencing data.

The close relationship between the two species was displayed by the high similarity shared between their *de novo*-assembled transcriptomes. Both genotypes had similar percentages of the filtered raw genes annotated, and the top five BLAST hits were the same for both. The standard for gene functionality descriptions is gene ontology (GO), and the role of genes in any organism is well described in classified, distinct, common GO terms (Ashburner et al., 2000). Through the use of FunctionAnnotator (Chen et al., 2017), similar percentages of the annotated genes in both genotypes were mapped to GO terms and GO biological process (GO-BP) terms identified in both genotypes again remained similar.

### Differential Gene Regulation in Response to Shade Is Stage-Dependent in *L. orientalis* and *L. culinaris*

It has been reported that regulatory changes likely play a more important role than coding changes in closely related species of both animals and plants (Jones et al., 2012; Ng et al., 2019). Lupa and BGE 016880 shared a high degree of similarity in their transcriptomes; however, gene expression levels in response to shade were quite different between them. The overall DEGs identified in BGE 016880 were consistently double when compared to those in Lupa under the same condition. This clearly shows there is a genotypic/species difference between BGE 016880 and Lupa in response to low R/FR conditions. A GO-BP enrichment analysis to classify the functions of the DEGs provided further insight into differential gene regulation in response to shade between BGE 016880 and Lupa.

The top enriched GO-BP terms among the upregulated DEGs at the T1 stage under shade were all from Lupa and included glucosinolate biosynthetic and metabolic processes, as well as indole-containing compound biosynthetic and metabolic processes. Among these were glucosinolates, a class of secondary metabolites that play significant roles in plant response to different abiotic stresses, especially plant defense to reduce the effects of pathogen attack (Martínez-Ballesta et al., 2013; Burow and Halkier, 2017). Salicylic acid is one of the central factors regulating plant defenses (Pieterse et al., 2012; Li et al., 2019), and multiple studies have reported an interdependent relationship between salicylic acid and glucosinolate in plant responses toward cold stress and herbivore attack (Byun et al., 2009; War et al., 2012). Other than auxin, other indole-containing compounds include defense and scent-related metabolites that have been reported to play important roles in plant fitness-related activities such as pollinator attraction and herbivore repulsion (Cna’ani et al., 2018). The synthesis of indole glucosinolates improved disease resistance of *Arabidopsis* against a fungal pathogen *Plectosphaerella cucumerina* (Sanchez-Vallet et al., 2010; Frerigmann et al., 2016). In addition, auxin has been shown to be an important regulator of SAS in the model species *Arabidopsis* and legumes such as *Lotus japonicus* (Ueoka-Nakanishi et al., 2011; Iglesias et al., 2018).

It was a different picture, however, when looking at the significantly enriched GO-BP terms within the downregulated DEGs at the T1 stage. One of the main terms, shared by both genotypes, relates to the endoplasmic reticulum. This organ is considered the gatekeeper of the secretory pathway in protein biosynthesis and helps to maintain the spatial organization and distribution of other organelles (Stefano and Brandizzi, 2018). Downregulation of these processes in plants exposed to shade might well affect cellular homeostasis and plant growth. Apart from that, the top enriched biological processes in downregulated DEGs at the T1 stage were all from BGE 016880. These enriched processes included several that were related to defense and immune responses such as “response to chemical,” “defense response to other organism,” and “regulation of defense response.”

Gene expression results could be very different because the tissues collected for gene expression came from distinct developmental time points. The leaf samples in the T1 stage used in this study were collected 2 weeks after emergence when both BGE 016880 and Lupa were at the vegetative growth stage. However, subsequent stages (T2–T5) were marked with the transition to reproductive growth for BGE 016880 and Lupa at different sample collection points. Therefore, group means of all five stages were used to look at the overall low R/FR-induced shade responses in these two genotypes. DEGs from all five stages combined had more enriched biological processes shared by both BGE 016880 and Lupa. However, processes that related to defense response and immune response at the T1 stage were still enriched among the downregulated DEGs from all five stages in BGE 016880 only. The differences in enriched GO-BP terms between the T1 stage and all five combined stages showed stage-dependent regulation existed in both genotypes, and gene regulatory differences were likely to be responsible for different responses of both genotypes toward low R/FR-induced shade.

### Shade Promotes Shoot Elongation in Both Genotypes While Reducing Defense Response Only in Wild *L. orientalis*

Under shade conditions, gibberellin (GA) plays an important role in stimulating stem elongation in plants and increased GA biosynthesis and signaling leads to increased elongation (Colebrook et al., 2014; Li et al., 2020). Multiple GA biosynthesis GA 20-oxidase genes were upregulated in both BGE 016880 and Lupa, with two of these genes shared by both genotypes (*LcGA20oxC*, Lcu.2RBY.2g037160, and *LcGA20oxG*, Lcu.2RBY.3g057140). GASA (GA-stimulated *Arabidopsis*) and GAST (GA-stimulated transcripts) genes in the GA signaling pathway are mostly upregulated by GA to influence a variety of processes including stem elongation (Zhang and Wang, 2017). GASA/GAST family members were identified in both BGE 016880 and Lupa as DEGs with one gene (Lcu.2RBY.6g048590) shared by both genotypes. Brassinosteroids (BRs) are plant hormones that promote elongation, and genes involved in the biosynthesis of BR (Bancoş et al., 2002) were also identified in both BGE 016880 and Lupa as upregulated DEGs, with one gene shared by both genotypes (Lcu.2RBY.6g004710).

**FIGURE 5:**
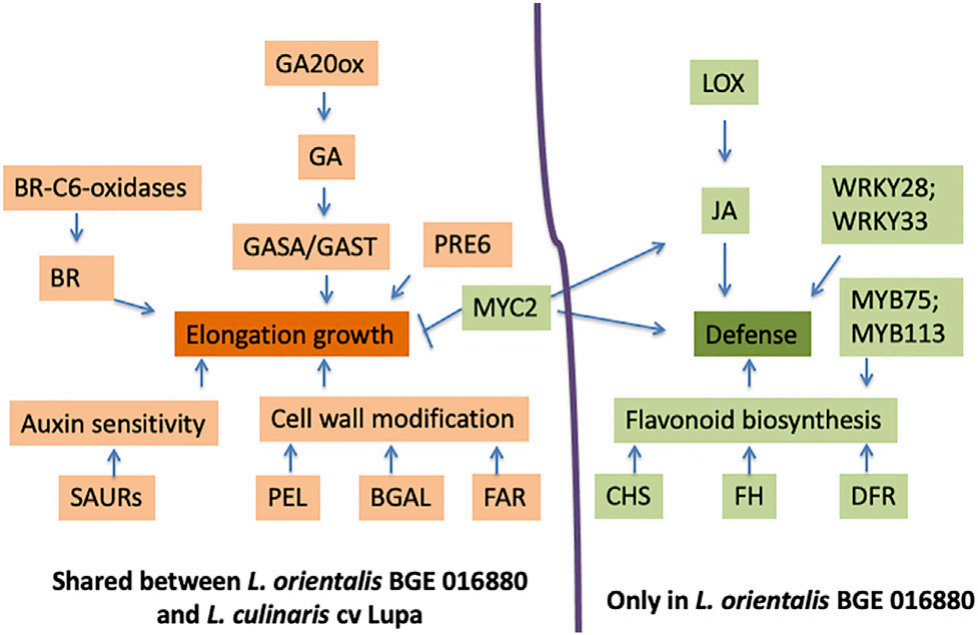
Model for roles and interactions of genes on elongation growth and defense response under low R/FR-induced shade conditions for both *L. orientalis* BGE 016880 and *L. culinaris* cv. Lupa. This model summarizes the major results from this study, and the hypothetical interactions are based on previous network studies on *Arabidopsis* and other crops (Carriedo et al., 2016; Ballaré and Pierik, 2017; Fernández-Milmanda and Ballaré, 2020). Arrows indicate a promoting interaction, and a T-end indicates an inhibiting interaction. Genes/biological processes in orange refer to upregulation, while green refers to downregulation.

Auxin signaling plays an important role in promoting stem growth and inhibiting branching, both of which are typical shade avoidance responses (Iglesias et al., 2018). Several auxin response genes such as small auxin-up RNAs *(SAURs)* were identified as upregulated DEGs in both BGE 016880 and Lupa, with two *SAURs* (Lcu.2RBY. 4g041680 and 4g044230) shared by both genotypes. The elongation response in *Arabidopsis* under shade was promoted by cell wall modification, particularly a loosening process (Sasidharan et al., 2010). Cell wall loosening involves the action of multiple proteins such as expansins, endoglucanases, xyloglucan endotransglycosylase/hydrolase (XTH), β-galactosidases (BGAL), pectate lyases (PEL), and pectin methylesterases (PEM) (Cosgrove, 2016; Leng et al., 2017; Moneo-Sánchez et al., 2019). Multiple BGAL and PEL genes were among the upregulated DEGs in both BGE 016880 and Lupa. The coordinated action of gibberellin (GA), brassinosteroid (BR), and auxin biosynthesis and signaling pathways, as well as the involvement of cell wall modification enzymes, promoted elongation responses in both BGE 016880 and Lupa under shade (Figure 5 and Table 2).

**TABLE 2:** DEGs and their corresponding gene numbers from the *L. culinaris* cv. CDC Redberry reference genome (v2.0) were used for the proposed model in Figure 5 regarding their roles and interactions on elongation growth and defense response under low R/FR-induced shade conditions for both *L. orientalis* BGE 016880 and *L. culinaris* cv. Lupa.

Gene family	BGE_T1	Lupa_T1	BGE_LH	Lupa_LH
GA 20-oxidase (GA20ox)	3g057140; 2g037160	3g057140	3g057140; 2g037160	3g057140; 2g037160; 7g014320
GA-stimulated *Arabidopsis*/GA-stimulated transcript (GASA/GAST)	7g043100; 6g048590; 6g002160	6g048590		6g002160
Cytochrome P450 family brassinosteroid oxidase (BR-C6-oxidases)	5g009500; 6g004710	6g004710		
Small auxin-up RNAs (SAURs)	4g044230; 4g041680	4g044230; 4g041680		4g041680
Pectate lyase (PEL)	7g058730; 1g010330	7g058730; 1g010330		
Beta-galactosidase (BGAL)	2g022960; 2g093770	2g022960; 7g004020		
bHLH TF (PRE6)	7g033720	7g033720		
Gland-specific fatty acyl-CoA reductase (FAR)	4g027410; 4g027370		4g027410; 4g027370	4g027370
bHLH TF (MYC2)	7g029860	7g029860	7g029860	
Lipoxygenase (LOX)	7g006280; 4g036980		4g036980	
Chalcone synthase (CHS)	3g046440; 5g001660		3g046440; 5g001660	
Flavonoid 3′,5′-hydroxylase (FH)	3g016660		3g016660	
Dihydroflavonol 4-reductase (DFR)	5g073610		5g073610	
MYB TF (*MYB75* and *MYB113*)	5g054070; 5g054150		5g054150	
WRKY TF (*WRKY28* and *WRKY33*)	4g062520; 3g029190		4g062520; 3g029190	

Jasmonic acid (JA) and salicylic acid (SA) pathways play key roles in the coordination of plant defense and the formation of the hormonal immune system (Lemarié et al., 2015; Betsuyaku et al., 2018). Perception of low R/FR signals activates shade avoidance responses and reduces the expression of defenses against pathogens and insects. Low R/FR-induced shade increased infection of pathogens such as *Botrytis cinerea* and *Pseudomonas syringae* in *Arabidopsis* (Cerrudo et al., 2012; De Wit et al., 2013), and the seriousness of plant disease is noticeably increased under high population density in agricultural settings (de Souza Jaccoud-Filho et al., 2016). Immune response was weakened in FR-treated *Arabidopsis* plants because of the corresponding decreased response to jasmonic acid and salicylic acid (Ballaré, 2014; Fernández-Milmanda and Ballaré, 2020).

A series of biological processes that are related to defense response and immune response were enriched in downregulated DEGs in *L. orientalis* BGE 016880 but not in *L. culinaris* cv. Lupa. JA is one of the main players in plant defense and immune responses, and multiple genes involved in JA biosynthesis were identified as downregulated DEGs in BGE 016880, including lipoxygenase (Lcu.2RBY.4g036980). Flavonoids are main players in plant defense system as well (Treutter, 2005; Ali and McNear, 2014; Lu et al., 2017), and multiple genes involved in flavonoid biosynthesis, including chalcone synthase (CHS), flavonoid hydroxylase (FH), and dihydroflavonol reductase (DFR), were among the downregulated DEGs in BGE 016880. CHS is not only important in flavonoid biosynthesis but also takes part in the SA defense pathway (Dao et al., 2011). We speculate that SAS in wild lentil accession BGE 016880 is associated with reduced defense response (Figure 5 and Table 2). From an adaptive point of view, the suppression of defense responses will probably save the resources for plants and give the priority to elongation and other SAS-related traits, which will maintain the competitiveness of plants. However, this response may have been unintentionally reduced in cultivated crops through breeding efforts where yield and disease resistance are simultaneously selected at high density (Carriedo et al., 2016). Our results support this theory since the wild accession *L. orientalis* BGE 016880 showed distinct suppression of defense-related biological processes with downregulation of related genes under shade conditions, in contrast to the cultivated type *L. culinaris* cv Lupa**.**

### Transcription Factors in Shade Responses in Lentils

Transcription factors (TFs) have been shown to play a major role in light-regulated transcriptional networks such as SAS (Jiao et al., 2007), and a large number of shade-induced TFs have been identified and functionally characterized in *Arabidopsis*. These include family members of bHLH, WRKY, MYB, and homeobox genes (Hornitschek et al., 2009; Zhou et al., 2014; Galvāo et al., 2019; Buti et al., 2020). In this lentil study, the number of TF DEGs was more than doubled in BGE 016880 than Lupa, at the T1 stage and across the five stages.

Within the differentially expressed TF, there was a high presence of bHLHs at both the T1 stage and all five stages in both BGE 016880 and Lupa. The bHLH family is one of the largest TF groups in plants and has various roles in plant development, especially in developmental adaptation to light signals (Hao et al., 2012; Buti et al., 2020). Overexpression of members within the PRE subfamily of bHLHs resulted in developmental changes linked with increased gibberellin responses such as elongated hypocotyls and early flowering (Lee et al., 2006). *PRE1*, *PRE2*, *PRE4*, and *PRE6*, all members of the PRE subfamily, have shown strong upregulation under low R/FR and promote elongation growth (Kohnen et al., 2016; Gommers et al., 2017). One of the differentially expressed bHLHs (Lcu.2RBY.7g033720) from the T1 stage that was upregulated in both BGE 016880 and Lupa under low R/FR is a homolog of *PRE6.* This could be related to the similar elongation responses by the two genotypes under shade condition.

Another bHLH family member (Lcu.2RBY.7g029860), which was downregulated in both BGE 016880 and Lupa, is a homolog of *MYC2*. *MYC2* has been shown to be a central player that integrates different environmental and developmental signals and contributes to the regulation of GA and JA pathways (Pireyre and Burow, 2015). *MYC2* promotes the activation of *HY5* to repress cell elongation-related genes involved in seedling growth (Yi et al., 2020). Downregulation of the *MYC2* gene could well be linked with the elongation responses in both BGE 016880 and Lupa. *MYC2* has also been shown to be the center of JA signal pathways to activate the JA response (van Moerkercke et al., 2019; Zander et al., 2020).

The MYB family is another large group of TFs involved in the regulation of multiple plant development processes and considered the key regulator of the flavonoid pathway (Stracke et al., 2007; Ambawat et al., 2013). Increased expressions of *MYB11*, *MYB12, MYB75, MYB111, MYB113*, and *MYB114* have been shown to increase the activity of the flavonoid biosynthesis pathway (Stracke et al., 2007; Ali and McNear, 2014; Onkokesung et al., 2014). There were multiple differentially expressed MYBs in BGE 016880 at the T1 stage and all five stages as compared to only a couple in Lupa. The majority of these in BGE 016880 were downregulated, and two of them (Lcu.2RBY.5g054070 and Lcu.2RBY.5g054150) are homologs of *MYB75* and *MYB113*. The downregulation of these MYBs could be related to the downregulation of flavonoid biosynthesis genes in BGE 016880, while this pathway was not affected in Lupa (Figure 5 and Table 2).

Large numbers of differentially expressed WRKYs were also identified in BGE 016880 but not in Lupa, and almost all of them were downregulated. WRKY TFs have various roles in plants including in disease resistance, abiotic stress response, and hormone-controlled biological processes (Bakshi and Oelmüller, 2014). However, the most prominent role of WRKY TFs is probably the regulation of plant defense and stress responses. WRKYs such as *WRKY3, WRKY4, WRKY23, WRKY28, WRKY33,* and *WRKY50* were involved in resistance to multiple bacterial or fungal pathogens in multiple plant species (Lai et al., 2008; Jing et al., 2009; Wu et al., 2011; Birkenbihl et al., 2012; Hussain et al., 2018). Soybean *WRKY* genes were responsive to salicylic acid and promoted resistance to soybean cyst nematode (Yang et al., 2017). Peanut *WRKY* genes were involved in drought stress and disease responses as well (Zhao et al., 2020). Homologs of WRKYs such as *WRKY3, WRKY23, WRKY28, WRKY33*, and *WRKY50* were among the downregulated TFs at the T1 stage, with *WRKY28* and *WRKY33* (Lcy.2RBY.3g029190 and 4g062520) shared between the T1 stage and all five stages in BGE 016880. Good representation of WRKY transcription factors among the DEGs and the general trend of downregulation of these WRKYs may contribute to a reduced defense response in *L. orientalis* BGE 016880 under shade conditions.

## Conclusion

Our study examined low R/FR-induced shade responses of lentils using a cultivated genotype and a wild accession of *L. orientalis*. Despite the high similarity between the transcriptomes and gene ontologies, DEG analysis and GO enrichment analysis identified both conserved and divergent regulations in response to shade. The differences in known shade-responsive genes, transcription factors, and their respective enriched GO biological processes indicated the divergence of these two species with respect to shade responses. Of note is the reduction in biotic and abiotic stress-related genes observed in the wild accession but not the cultivated one, suggesting that breeders may have inadvertently balanced shade responses with stress tolerance in cultivated lentils. This study will help deepen the understanding of shade responses in pulse crop species and their wild relatives and develop genetic strategies for crop improvement in response to changes in light environments. The intercorrelated network between typical SAS such as flowering time and shoot elongation and the defense pathway further proved the necessity to use an integrated approach when working with SAS in crop plants.

## Data Availability

The raw RNAseq data from *L. culinaris* cv. Lupa and *L. orientalis* BGE 016880 have been submitted to the NCBI Sequence Read Archive with ID PRJNA693582.
