# Supplementary material for: RNA-Seq and Gene Ontology Analysis Reveal Differences Associated With Low R/FR-Induced Shade Responses in Cultivated Lentil and a Wild Relative
Source: Front Genet. 2022 Jun 20;13:891702. doi: 10.3389/fgene.2022.891702 (PMC9251359; doi:10.3389/fgene.2022.891702)
Supplement: Supplementary file 1 [file DataSheet2.pdf]

```
#!/usr/bin/perl

use strict;
use warnings;
use Data::Dumper;

my $SAM_file = $ARGV[0];
my $deg_list = $ARGV[1];
my $usage = "./convert_TRINITY_DEG_list.pl input.SAM
input.DEG_list";

unless (defined $SAM_file) {
        die "ERROR: Either a SAM file wasn't specified or it can't
be found. Usage: $usage";
}
unless (defined $deg_list) {
        die "ERROR: Either a DEG list wasn't specified or it could
not be found. Usage: $usage";
}

my @splitline;
my @splitread;
my %readnames;
my ( $readname, $gene, $trinityname );

# Store our read names from the SAM file into a hash
# Assumes a non-header SAM file
open SAMFILE, $SAM_file or die "ERROR: Unable to open $SAM_file\n";

while (my $alignment = <SAMFILE>) {

        chomp $alignment;
        @splitline = split(/\t/, $alignment);
        $readname = $splitline[0];
        # Alter the readname since the DEG list leaves off the
"_i#"
        $readname =~ s/_i[0-9]*$//g;
        $trinityname = $readname;
        @splitread = split(/_/, $readname);
        $gene = $splitread[0];
        $trinityname =~ s/$gene/TRINITY/;
        ## There might already be a sequence ID with this name if
the read aligned to more than one gene,
        ## so treat this as a hash of arrays
        ## Since the DEG format is less specific for its Trinity
names, make sure we do exclude if we have this exact gene/TRINITY
name combo already
        unless (exists $readnames{$trinityname} && (grep /$gene/,
@{$readnames{$trinityname}})) {
                push @{ $readnames{$trinityname} }, $gene;
        }
}
close SAMFILE;
```

```
my $genename;

# Loop through the DEG list IDs and change any target names that
were in the SAM file
open DEG, $deg_list or die "ERROR: Unable to open $deg_list\n";

while (my $line = <DEG>) {
        foreach my $key (keys %readnames) {
                if ( $line =~ /$key\s/ ) {
                        my $newtarget;
                        foreach $genename ( @{$readnames{$key}} )
{
                                $newtarget .= $genename . "_";
                        }
                        $line =~ s/TRINITY_/$newtarget/g;
                        last;
                }
        }
        print $line;
}

close DEG;
```
