# Supplementary material for: RNA-Seq and Gene Ontology Analysis Reveal Differences Associated With Low R/FR-Induced Shade Responses in Cultivated Lentil and a Wild Relative
Source: Front Genet. 2022 Jun 20;13:891702. doi: 10.3389/fgene.2022.891702 (PMC9251359; doi:10.3389/fgene.2022.891702)
Supplement: Supplementary file 4 [file DataSheet1.docx]

Supplementary Material

RNAseq and Gene Ontology Analysis Reveal Differences Associated with Low R/FR induced Shade Responses in Cultivated Lentil and its Wild Relatives

**Hai Ying Yuan^1, 2^, Carolyn T. Caron^1^, Albert Vandenberg^1^, Kirstin E. Bett^1,^ ***

^1^Department of Plant Sciences, University of Saskatchewan, Saskatoon SK Canada

^2^Current address: Aquatic and Crop Resource Development Research Center, National Research Council of Canada, Saskatoon SK Canada

*** Correspondence:**Kirstin E. Bett
[k.bett@usask.ca](mailto:k.bett@usask.ca)

Supplementary Table S1. Summary statistics of differentially expressed transcription factor (TF) classes in both *L. orientalis* BGE 016880 and *L. culinaris* cv. Lupa. TFs were assigned using Mercator pipeline (Lohse *et al.*, 2014; Schwacke *et al.*, 2019) from *de novo* assembled transcriptomes of both genotypes and then used to identify differentially expressed TFs from DEG lists.

| Differentially expressed TF class | *L. orientalis* BGE 016880 | | *L. culinaris* cv. Lupa | |
| --- | --- | --- | --- | --- |
|  | T1 | LH | T1 | LH |
| bHLH | 13 | 11 | 6 | 4 |
| MYB | 13 | 6 | 2 | 2 |
| WRKY | 10 | 6 | 1 | N/A |
| AP2/ERF | 9 | 6 | 3 | 2 |
| Homeobox | 6 | 1 | 1 | 1 |
| MADS/AGL | 4 | 7 | N/A | 4 |
| NAC | 5 | 2 | N/A | 1 |
| Other TFs | 24 | 4 | 8 | 6 |
| Total TF DEGs | 84 | 43 | 21 | 20 |


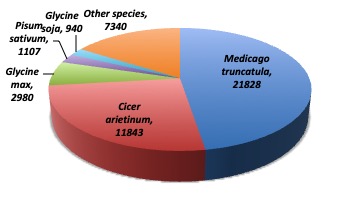

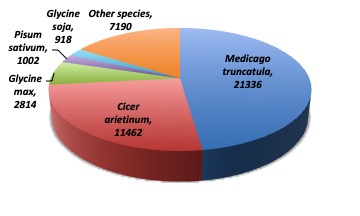


**A**

**B**

Supplementary Fig. S1 Taxonomy distribution of top BLAST hits for individual genes and the respective gene counts from *de novo* assembled transcriptomes of *L. orientalis* BGE 016880 (A) and *L. culinaris* cv. Lupa (B).


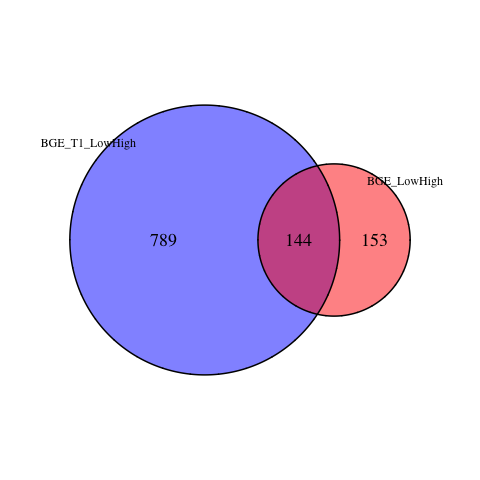

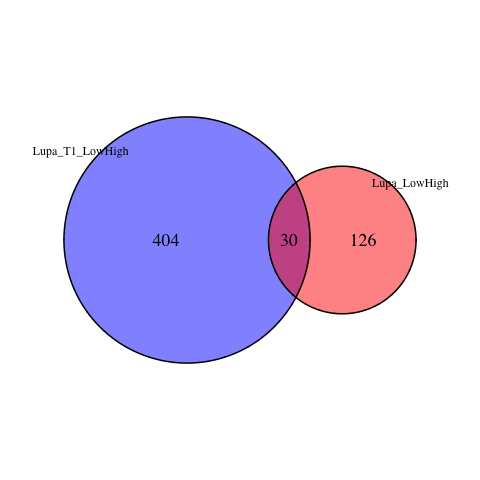


**B**

**A**

Supplementary Fig. S2 Venn Diagrams shown numbers of unique and common differentially expressed genes (DEGs) between T1 stage (T1_LowHigh) and all five growth stages (LowHigh) under low R/FR induced shade condition for both *L. orientalis* BGE 016880 (A) and *L. culinaris* cv. Lupa (B)


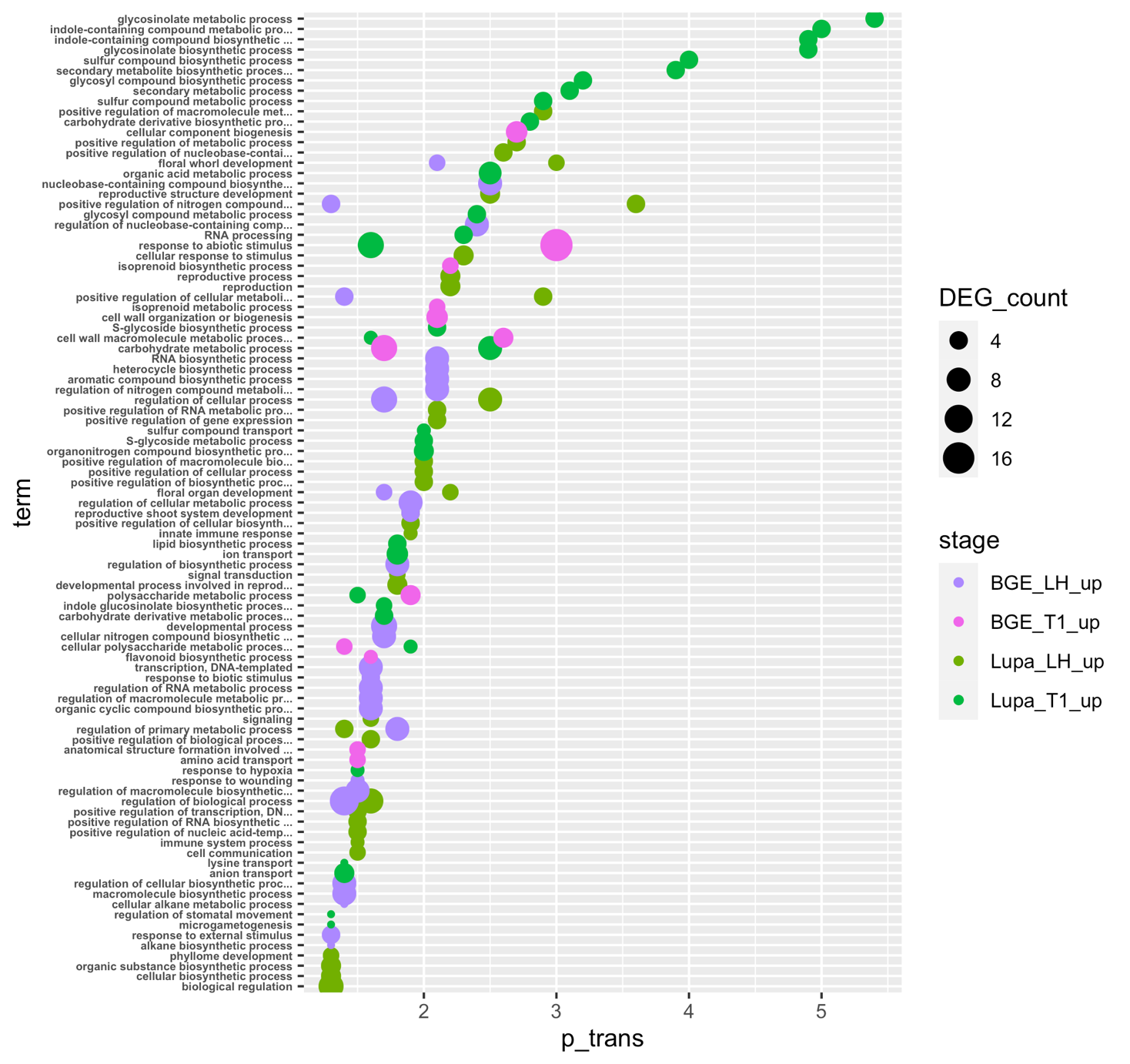


Supplementary Fig. S3 Significant enriched GO: BP terms in up-regulated DEGs from T1 stage and all five stages of *L. orientalis* BGE 016880 and *L. culinaris* cv Lupa under low R/FR induced shade condition. TopGO (version2.22) is employed and Fisher’s exact tests were performed to find the significant enriched GO terms for the input gene set. P-value ≤ 0.05 was considered as significant. X-axis (p_trans) shows –log (P-value), while Y-axis shows significant enriched GO terms in Biological Process. DEG-count means the number of up-regulated DEGs within each enriched GO-BP term.


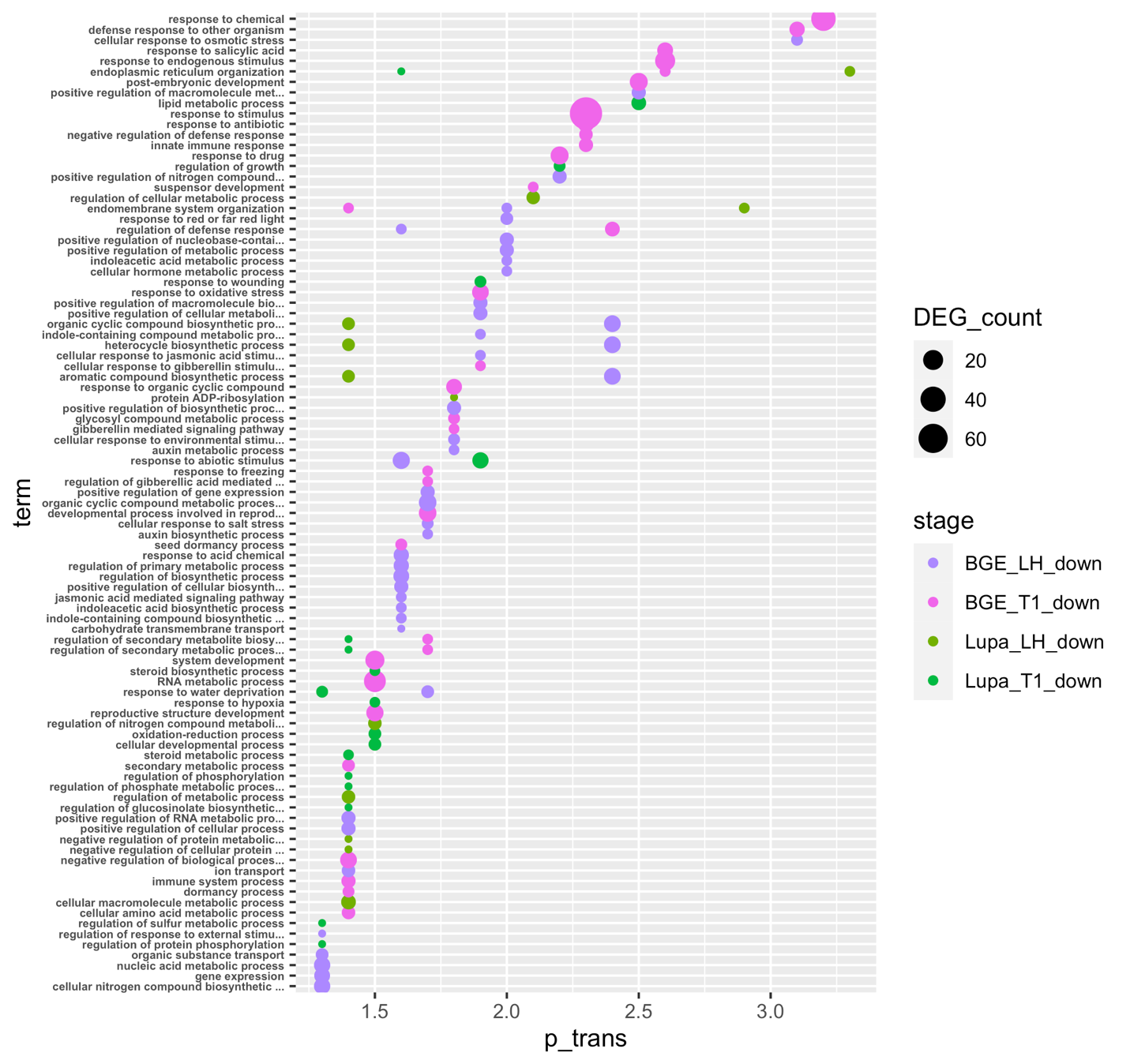


Supplementary Fig. S4 Significant enriched GO-BP terms in down-regulated DEGs from T1 stage and all five stages of *L. orientalis* BGE 016880 and *L. culinaris* cv Lupa under low R/FR induced shade condition. TopGO (version2.22) is employed and Fisher’s exact tests were performed to find the significant enriched GO terms for the input gene set. P-value ≤ 0.05 was considered as significant. X-axis (p_trans) shows –log (P-value), while Y-axis shows significant enriched GO terms in Biological Process. DEG-count means the number of down-regulated DEGs within each enriched GO-BP term.
